# Psychometric validation of the Ego-undercontrol scale in Chinese college students

**DOI:** 10.3389/fpsyg.2025.1597994

**Published:** 2025-09-11

**Authors:** Bibo Mo, Xiaoshi Liu, Yezi Chen, Lijie Ren, Xiaofeng Wang

**Affiliations:** ^1^Teaching Office of Psychology, Zhejiang Wanli University, Ningbo, China; ^2^Mental Health Education and Guidance Centre, Zhejiang Wanli University, Ningbo, China; ^3^Department of Psychology, Yangzhou University, Yangzhou, China; ^4^School of Urban Governance and Public Affairs, Suzhou City University, Suzhou, Jiangsu, China; ^5^Mental Health Education Center, Shanghai Customs University, Shanghai, China; ^6^Student Mental Health Education Centre, Shanghai University of Political Science and Law, Shanghai, China

**Keywords:** ego-control, psychometric properties, factor structure, validation, Chinese university students

## Abstract

Ego-control is considered a vital element of ego functioning. This study aimed to explore the factor structure and psychometric properties of the Chinese version of the Ego-undercontrol Scale (EUC-C) in Chinese college students. A sample of 1,823 participants from three general universities in Shanghai, China (mean age = 19.70, SD = 1.42, 44.2% male individuals), completed the survey. A shorter version of the EUC-C was developed. Confirmatory factor analysis (CFA) validated a two-factor structure of the EUC-C, comprising impulse and inhibitory behaviors. In addition, the EUC-C showed adequate validity and reliability. In conclusion, the results showed that the EUC-C has acceptable psychometric properties, making it a valid and reliable instrument for assessing ego-control among Chinese university students.

## Introduction

Research on self-control has grown steadily over recent decades, resulting in a wealth of theoretical and empirical studies that enhance our understanding of the topic (e.g., Duckworth et al., 2016; Duckworth et al., 2019). Many studies have emphasized the benefits of high self-control (e.g., Duckworth et al., 2019); however, it should be noted that high self-control can also have negative effects. For instance, children with high self-control have reported more internalizing problems (e.g., Volbrecht and Goldsmith, 2010). In addition, individuals with a high level of self-control are also more likely to focus solely on the achievement of personal goals, which may be detrimental to their well-being (McGregor and Little, 1998). From a theoretical standpoint, Kremen and Block (1998) argued that the failure to distinguish between maladaptive and adaptive impulse control is a key conceptual flaw. Furthermore, Block (2002) noted that the unqualified positive use of the term “self-control” fails to acknowledge that inhibitory control, although usually adaptive, can also influence behavior by making it rigid, inexpressive, routine, emotionally flat, and focused on excessive postponement of emotional gratification—traits that are not uncommon in individuals with a high level of self-control.

Self-control in a psychological context is substantially distinct from ego-control. *Self-control* involves suppressing impulses that are socially unacceptable and ineffective in daily life (Kremen and Block, 1998). However, this definition of self-control carries value-laden connotations and is, in actual fact, somewhat illogical, implying that adaptation requires an individual to move in the direction of becoming overly controlling. In fact, this conceptualization of self-control implies that undercontrol is necessarily undesirable, both socially and cognitively (Kremen and Block, 1998). Thus, the implications of the concept of self-control are ambiguous (Kremen and Block, 1998). For example, the concept of self-control does not distinguish between adaptive impulsive behavior (behavior that matches the situation, e.g., expressions of warmth) and maladaptive impulsive behavior (behavior that does not match the situation, e.g., unrestrained aggressive behavior).

### Concept of ego-control

To address the ambiguity surrounding self-control, Jack Block and Jeanne H. Block developed the concept of ego-control (Block, 1950; Block, 1951). As a personality construct, ego-control affects a considerable array of consequential life outcomes, such as cognition (e.g., Block, 2005), marijuana use (e.g., Shedler and Block, 1990), adaptive functioning (e.g., Cicchetti and Rogosch, 2007), and mental health problems (e.g., Bohane et al., 2017). Ego-control refers to “the individual’s modal or characteristic response to behavioral or attentive impulses” (Block and Block, 2006, p. 317), and it ranges from undercontrol to overcontrol. According to Block and Block (2006), an individual who is relatively expressive or attentive to internal pushes and pulls (e.g., immediate and direct expression of behaviors) can be described as undercontrolled; in contrast, individuals who are relatively restrictive in their behavioral or attentional impulses (e.g., excessive delayed gratification) are referred to as overcontrollers. A growing body of literature indicates that both undercontrol and overcontrol in individuals are related to an increased incidence of health problems (for reviews, see Alessandri and Vecchione, 2017; Bohane et al., 2017). Specifically, undercontrolled individuals tend to show externalizing problems (e.g., aggressive behavior), whereas overcontrolled individuals tend to show internalizing problems (e.g., depression).

### Measurement of ego-control

Jack Block developed the *ego-undercontrol scale* (EUC; Letzring et al., 2005) to evaluate ego-control in individuals. This self-report measure comprises 37 items, each of which is rated using a four-point scale ranging from 1 (*disagree very strongly*) to 4 (*agree very strongly*). Letzring et al. (2005) reported that Cronbach’s alpha of the original scale was only 0.63 and confirmed that the measure had a unifactorial structure. To the best of our knowledge, only two studies have further developed the EUC, revising it to achieve adequate validity and reliability: one study translated the scale into Swedish (Isaksson et al., 2021), while the other translated into Spanish for use in an Argentinian context (Hess and Mesurado, 2021). The Swedish version of the EUC (EUC-13; Isaksson et al., 2021) includes 13 items and comprises 3 dimensions: Uninhibited behavior (e.g., “When I get bored, I like to stir up some excitement”), planful conscientious behavior (e.g., “I like to stop and think things over before I do them”), and socially restrained behavior (“I am easily downed in an argument”). Unfortunately, Isaksson et al. (2021) did not clarify the meaning of each dimension, and it should be noted that the internal reliability for the socially restrained behavior subscale was only 0.51. Furthermore, the factor loading of the EUC-13’s item 11 (“I am easily downed in an argument”) was 0.26 in Isaksson et al.’s (2021) study, which is below 0.4. The results suggest that the EUC-13 may also fail to achieve satisfactory psychometric properties.

The Spanish version of the EUC (Hess and Mesurado, 2021) contains 14 items and measures 2 dimensions: Behavioral impulsiveness (e.g., “I tend to buy things on impulse”) and cognitive impulsiveness (e.g., “I become impatient when I have to wait for something”). Behavioral impulsiveness refers to a lack of control over impulses related to physical behaviors. Cognitive impulsiveness reflects one’s ability to stop and think about a situation before taking action (Hess and Mesurado, 2021). The factor loading of item 18 (“I like to flirt”) was 0.23 in Hess and Mesurado’s (2021) study, which is again below 0.4; however, the results indicate that the psychometric properties of the Spanish version of the EUC also fail to meet acceptable standards. Furthermore, while the EUC-13 and the Spanish version of the EUC both measure multiple dimensions, the studies that introduced these scales lack theoretical support for the identified dimensions. To return to ego-control theory (Block and Block, 1980), if ego-control refers to one’s ability to express or inhibit their impulses, with one end of the trait spectrum representing undercontrol and the other representing overcontrol, it is logical to conclude that the EUC and its derivatives measure only two dimensions: impulsive behavior and inhibitory behavior.

### The current study

To the best of our knowledge, no scale has been developed as of yet to evaluate ego-control in a Chinese context. Therefore, to further research ego-control in China, as well as to extend Block and Block’s theories about ego function, this study aimed to translate and validate the EUC into Chinese. As exploratory structural equation modeling (ESEM; Asparouhov and Muthén, 2009; Marsh et al., 2014) incorporates exploratory factor analysis into the framework of structural equation modeling, ESEM was utilized to assess the factorial validity of the newly developed measure.

We first translated the English-language EUC into Chinese to create the Chinese version of the EUC (EUC-C), after which we tested its psychometric properties using a non-clinical Chinese sample. We then assessed its factorial validity using ESEM and confirmatory factor analysis (CFA). We hypothesized that the EUC-C would have two dimensions, measuring impulsive behavior and inhibitory behavior. Finally, we assessed the construct validity of the EUC-C by investigating its correlations with other constructs. We hypothesized that ego-undercontrol would be positively related with loneliness and depression and negatively associated with self-esteem and life satisfaction.

## Methods

### Participants

A sample of 1,823 freshmen, sophomores, and juniors was recruited (*M_age_* = 19.70; *SD* = 1.42; 44.2% males) from three general universities in Shanghai. There were 944 freshmen (42.4% male), 686 sophomores (47.8% male individuals), and 193 juniors (40.4% male individuals).

### Procedures

The questionnaire was administered to the students with the help of their counselors in a classroom setting. All participants who took part in this study provided their consent to participate. Each participant completed a paper-and-pencil questionnaire in class and submitted it upon completion. The study was reviewed and approved by the ethics committee of the corresponding author’s institution.

### Measure

#### Ego-undercontrol

The *Ego-undercontrol Scale* (EUC; Letzring et al., 2005) was developed by Professor Jack Block. The scale consists of 37 items and is scored on a 4-point Likert scale ranging from 1 (*strongly disagree*) to 4 (*strongly agree*). A total of 15 items were reverse-scored, and after converting the reverse-scored entries, higher scores represented higher levels of ego-undercontrol. First, the English version of the Ego-undercontrol Scale was translated into Chinese by two PhD students in psychology. Then, the Chinese version was translated back into English by two English teachers with PhDs. Second, Prof. Letzring, the main author of the original version, reviewed and revised the back-translated version of the Ego-undercontrol Scale and finally formed the Chinese version of the Ego-undercontrol Scale.

#### Self-esteem

Self-esteem was assessed using the *Rosenberg self-esteem scale* (RSES; Rosenberg, 1965). The scale consists of 10 items (e.g., “On the whole, I am satisfied with myself”). Each item is rated on a 4-point scale (1 = *strongly disagree*, 4 = *strongly agree*). This measure has previously been validated in samples of Chinese students (e.g., Gao et al., 2022). Cronbach’s alpha was 0.88.

#### Life satisfaction

We measured life satisfaction using the *Satisfaction with Life Scale* (SWLS; Diener et al., 1985). The scale contains 5 items (e.g., “In most ways, my life is close to my ideal”). Each item was rated on a 7-point scale ranging from 1 (*strongly disagree*) to 7 (*strongly agree*). This scale has previously been validated in samples of Chinese students (e.g., Bieda et al., 2019). Cronbach’s alpha was 0.85.

#### Loneliness

*The UCLA Loneliness Scale* (Version 3; Russell, 1996) is a 20-item questionnaire (e.g., “You often feel a lack of companionship.”) that uses a 5-point scale, ranging from 1 (*completely not true*) to 4 (*completely true*). This measure has previously been validated in samples of Chinese students (e.g., Ren et al., 2023). Cronbach’s alpha was 0.91.

#### Depression

Depression was assessed using the *Centre for Epidemiologic Studies Depression Scale* (CES-D; Radloff, 1977). The measure includes 20 items (e.g., ‘I feel depressed’). Each item is rated on a 4-point scale, ranging from 0 (*rarely or none of the time*) to 3 (*most or all of the time*). This measure has previously been validated in samples of Chinese students (e.g., Ren et al., 2023). Cronbach’s alpha was 0.92.

### Data analysis

We employed two methods for item analysis. First, we assessed the item-total correlation (ITC), which measures the relationship between the total score and individual item scores for the total dataset. An ITC value is considered appropriate when it is greater than 0.2, due to the fact that it is likely to be small in large samples (Kline, 2015; Yu et al., 2021). Second, we used the critical ratio (CR), which is a more sensitive and stable item discrimination index (Crocker and Algina, 1986; Yu et al., 2021). The total score for all items was ranked from the lowest to highest. The top 27% were classified as the high group, and the bottom 27% as the low group. An independent samples *t*-test was conducted between the high and low subgroups of the 37 items to find if there was a significant difference in the scores for each item.

The total sample was randomly divided into two subsamples using SPSS (version 22.0). We used Mplus (version 8.3) to conduct ESEM and CFA on the data. The first group (*N* = 911) was used for ESEM. Factor analyses were conducted using the Kaiser–Meyer–Olkin (KMO) test and Bartlett’s test to investigate sample adequacy. We used the oblique rotation to conduct the factor analyses. At the same time, a parallel analysis (O’Connor, 2000) was conducted using SPSS syntax to determine the upper limits of potential factor solutions, based on the comparison of actual eigenvalues and those generated through simulation using 1,000 samples of identical characteristics. The second group (*N* = 912) was used to confirm the factor structure.

Given that the EUC is a 4-point scale and not strictly a continuous variable, we used weighted least squares means and variance adjusted (WLSMV) estimation in Mplus software. This approach provides more accurate data results (Beauducel and Herzberg, 2006; Rhemtulla et al., 2012). To evaluate model fit, different fitting metrics were considered: the *χ*^2^/*df* goodness-of-fit index, the Comparative Fit Index (CFI), the Tucker–Lewis Index (TLI), Root Mean Square Error of Approximation (RMSEA), and standardized root mean square residual (SRMR). CFI and TLI values greater than 0.90 indicate acceptable fit (Hu and Bentler, 1999). RMSEA and SRMR values less than 0.08 indicate acceptable fit (Hu and Bentler, 1999). Model fit was assessed by comparing the goodness-of-fit indices between constrained and unconstrained measurement models, rather than comparing the respective chi-squared fit statistics. Both criteria have been widely used in previous studies. However, since Δχ^2^ is sensitive to the sample size, Cheung and Rensvold (2002) proposed a more practical criterion for comparing nested models, which is to consider changes in the goodness-of-fit indices. An increase of at least 0.010 in the CFI and the TLI or a decrease of at least 0.015 in the RMSEA indicates that one model outperformed the other model. In addition, given that missing data were minimal (<0.06%), we used full information maximum likelihood (FIML; Graham, 2009) to deal with missing values.

## Results

### ITC and item discriminability

Except for items 21 (*r* = 0.19, *p* < 0.001), 23 (*r* = −0.15, *p* < 0.001), 24 (*r* = −0.04, *p* = 0.09), 31 (*r* = 0.09, *p* < 0.001), 33 (*r* = 0.01, *p* = 0.77), 35 (*r* = 0.05, *p* = 0.02), 36 (*r* = 0.02, *p* = 0.32), and 37 (*r* = 0.03, *p* = 0.28), the ITC values of the other items were between 0.27 and 0.59, s*p*<0.001 (see Supplementary Table 1). In the item discriminant analysis, there were 486 and 493 participants in the low- and high-score groups, respectively. The independent samples *t-*tests indicated that, except for items 24 [*t*(967) = 0.86, *p* = 0.39], 33 [*t*(970) = 0.42, *p* = 0.67], 35 [*t*(971) = 1.96, *p* = 0.051], 36 [*t*(972) = 1.07, *p* = 0.28], and 37 [*t*(973) = 0.71, *p* = 0.48], the CR values of the other items were significant, s*p*<0.001.

In addition, in terms of content, items 21 (“In a group of people, I would not be embarrassed to be called on to start a discussion or give an opinion about something I know well”), 23 (“I am against giving money to beggars”), 24 (“It is unusual for me to express strong approval or disapproval of the actions of others”), 31 (“I keep out of trouble at all costs”), 33 (“I am easily downed in an argument”), 35 (“My conduct is largely controlled by the customs of those about me”), 36 (“It makes me uncomfortable to put on a stunt at a party even when others are doing the same sort of thing”), and 37 (“I find it hard to make small talk when I meet new people”) had low content discrimination validity among Chinese college students. Therefore, according to the above results, the items 21, 23, 24, 31, 33, 35, 36, and 37 were deleted.

### Exploratory factor analysis

In the first group (*N* = 911), the KMO test (0.91) and Bartlett’s test of sphericity (*χ*^2^/*df* = 21.88, *p* < 0.001) showed a good data fit for EFA. To decide the maximum number of factors, we performed a parallel analysis. The results showed that the maximum number of factors was four (see Table 1; Supplementary Figure 1). Subsequently, we used ESEM to evaluate one-factor, two-factor, three-factor, and four-factor models (see Supplementary Table 2). The ESEM results showed that, except for item 17, only the two-factor model had factor loadings greater than 0.30 for all items, and there were no cross-loading items (see Supplementary Table 3). More importantly, previous researchers (e.g., Bandalos and Gerstner, 2016; Lim and Jahng, 2019; Reio and Shuck, 2015) have suggested that the structure of a model should be consistent with the theory. Given that Block defined ego-control as the ability to express or inhibit impulses (Letzring et al., 2005), we finally extracted two factors: impulsive behavior and inhibitory behavior. Considering that the factor loadings were greater than 0.6, the structure of the factor was relatively stable and independent of the sample size (e.g., Beavers et al., 2013; Guadagnoli and Velicer, 1988). More importantly, the factor loadings were above 0.60, which indicated the unidimensionality of the measures (e.g., Asnawi et al., 2019; Mahfouz et al., 2020). This means that the items loaded on each dimension were relatively stable, and there was no cross-loading. Thus, in line with previous research (e.g., Awang, 2015; Wang et al., 2022), we removed items with factor loadings <0.60, starting with the lowest loading item and proceeding one item at a time, until there were no items with factor loadings <0.60. Finally, a total of 11 items were retained (see Table 2). Factor 1, named *impulsive behavior*, contained items 12, 13, 14, and 15. Factor 2, named *inhibitory behavior*, contained items 25, 26, 27, 28, 29, 30, and 32. The ESEM results showed a good model fit, *χ*^2^/df = 4.62, *p* < 0.001, CFI = 0.968, TLI = 0.948, RMSEA = 0.063, 90% CI = [0.053, 0.073], SRMR = 0.025 (see Supplementary Table 4).

**Table 1:** Parallel analysis.

Eigenvalues	Random means	Random 95th percentile	Real data
1	1.36	1.40	6.86
2	1.31	1.34	4.47
3	1.27	1.30	1.50
**4**	**1.24**	**1.27**	**1.30**
5	1.21	1.24	1.18
6	1.18	1.21	1.06

Bold values means the maximum number of factors was four.

**Table 2:** Standardized factor loadings.

Item	Factor 1	Factor 2
12	0.60*	0.07*
13	0.61*	−0.02
14	0.69*	−0.09*
15	0.79*	0.01*
25	0.01	0.76*
26	0.06	0.67*
27	0.07*	0.60*
28	−0.15*	0.79*
29	−0.07*	0.76*
30	−0.003	0.69*
32	0.001	0.77*

Items with factor loadings | ≥ 0.60| are in bold. *p < 0.05.

In addition, in terms of content, items 12 (“I do not always tell the truth”), 13 (“My way of doing things can be misunderstood or bother others”), 14 (“Sometimes I rather enjoy going against the rules and doing things I am not supposed to”), and 15 (“At times, I am tempted to do or say something that others would think inappropriate”) represented impulsive behaviors for Chinese college students. In terms of content, items 25 (“I like to stop and think things over before I do them”), 26 (“I do not like to start a project until I know exactly how to proceed”), 27 (“I finish one activity or project before starting another”), 28 (“I am steady and planful rather than unpredictable and impulsive”), 29 (“On the whole, I am a cautious person”), 30 (“I do not let too many things get in the way of my work”), and 32 (“I consider a matter from every viewpoint before I make a decision”) represented inhibitory behaviors for Chinese college students. Therefore, these items are consistent with the assumption of this study. Notably, compared to the original 37-item scale, the shortened 11-item version is more concise (i.e., it determines which items are impulsive behaviors and which items are inhibitory behaviors), easier to administer, and more cost-effective. This facilitates the advancement of research related to ego-control.

### Confirmatory factor analysis

In the second subsample (*N* = 912), a confirmatory factor analysis was performed to test the previously identified factor structure. The two-factor model fit was acceptable: χ^2^/*df* = 4.88, *p* < 0.001, CFI = 0.956, TLI = 0.944, RMSEA = 0.065, 90% CI = [0.057, 0.074], SRMR = 0.039. All standardized factor loadings for the CFA were significant (*p* < 0.001), ranging from 0.61 to 0.79 (see Supplementary Figure 2). Moreover, we examined the second-order two-factor and bi-factor models and found that the models did not converge with the data.

### Measurement invariance across sex

In the total sample (*N* = 1,823), the results of the measurement invariance tests across sex are shown in Supplementary Table 4. Measurement invariance was confirmed at all constructs, metrics, and scalar levels, with changes of less than 0.010 in CFI and TLI and less than 0.015 in RMSEA, compared to each model. The results suggested that ego-undercontrol scores had the same basic construct and measurement properties for both male and female participants (see Supplementary Table 5).

### Reliability analysis

Reliability tests were carried out on the total sample. Two months later, we randomly selected a class to conduct the reliability tests. The class consisted of freshmen (*N* = 55), including 24 male students. There was no evidence to suggest that this class was not representative. The Cronbach’s alpha coefficients for all items, the impulsive behavior factor, and the inhibitory behavior factor were 0.78, 0.77, and 0.88, respectively. Two-month test–retest reliabilities for all items, the impulsive behavior factor, and the inhibitory behavior factor in the sample (*N* = 55) were 0.74, 0.74, and 0.89, respectively.

### Criterion-related validity

As shown in Table 3, ego-undercontrol and impulsive behavior were significantly positively associated with loneliness, depression, and negative affect. However, they were significantly negatively associated with self-esteem, life satisfaction, and positive affect. In contrast, inhibitory behavior was significantly positively associated with self-esteem, life satisfaction, and positive affect, whereas it was significantly negatively associated with loneliness, depression, and negative affect. Finally, ego-undercontrol was significantly negatively associated with inhibitory behavior. There was no significant association between impulsive behavior and inhibitory behavior.

**Table 3:** Correlations between ego-undercontrol and criterion variables.

Variables	1	2	3	4	5	6	7
1. Ego-undercontrol	—						
2. Impulsive Behavior	0.55^***^	—					
3. Inhibitory Behavior	−0.85^***^	−0.03	—				
4. Self-esteem	−0.39^***^	−0.29^***^	0.28^***^	—			
5. Life Satisfaction	−0.23^***^	−0.36^***^	0.05^*^	0.39^***^	—		
6. Loneliness	0.34^***^	0.41^***^	−0.15^***^	−0.55^***^	−0.44^***^	—	
7. Depression	0.33^***^	0.37^***^	−0.16^***^	−0.52^***^	−0.36^***^	0.66^***^	—

^*^p < 0.05, ^***^p < 0.001.

## Discussion

The purpose of the present study was to evaluate the psychometric properties of the Ego-undercontrol Scale (EUC-C) among Chinese undergraduates. The results showed that the EUC-11 had acceptable reliability and validity. A two-factor structure model was supported in the Chinese undergraduate students, along with strong invariance across sexes. Taken together, the EUC-C can be used for evaluating ego-undercontrol.

Previous research has found that the EUC and its other varieties all have either a one-factor (Letzring et al., 2005) or a three-factor model structure (Isaksson et al., 2021). In the present study, the EUC-C was found to have a two-factor model structure. These differences could be due to differences in sample sizes. For example, Letzring et al. (2005) used a relatively small sample size of 188 individuals for their study. Insufficient sample sizes may have detrimental effects on the factor analysis process, resulting in unreliable and therefore invalid results (e.g., Osborne and Costello, 2004). Factor analysis requires a large sample (e.g., 400 or greater) to produce undistorted results (MacCallum et al., 1999; Norman and Streiner, 2014). Furthermore, Isaksson et al.’s (2021) study found that some items in the EUC-13 had low factor loadings below 0.6. More specifically, the factor loading of item 33 was 0.27 in Isaksson et al.’s (2021) study. However, according to Chin (1998), only items with factor loadings of at least 0.6 or higher would result in each measure accounting for 50% or more of the latent variable’s variance. Another possible explanation for the differences in the number of dimensions across various EUC measures could be cultural differences. In the current study, some items were shown to have low discriminative validity, such as item 17, “I would like to be a journalist.” This may be because in Chinese culture, there is no connection between becoming a journalist and ego-control. As the strength of certain items may not be consistent across various cultures, the EUC-C structure differs from that of the EUC, EUC-13, and the Spanish version of the EUC. It should be noted that the number of factors selected in previous research exploring the EUC measures (e.g., Isaksson et al., 2021) was data-driven and not theoretically supported, while in the current study, the two-factor model was identified based on the tenets of ego-control theory. The results of the present study also showed that the EUC-C had measurement invariance in configurations, metrics, and scales across sex among the Chinese college students.

Unexpectedly, the results of the current study showed an insignificant relationship between impulsive and inhibitory behaviors. According to ego-control theory (e.g., Kremen and Block, 1998), ego-control exists on a continuum, ranging from ego-undercontrol to ego-overcontrol. Certain concepts, such as internalizing or externalizing, are similar to the concepts of undercontrol and overcontrol (Block and Block, 2006). However, ego-control is a complex concept (Eisenberg et al., 2002, 2004), and it is important to note that externalizers are not necessarily undercontrollers and that internalizers are not necessarily overcontrollers (Block and Block, 1980). This is because the direction of motivational responses can be unrelated to the immediacy or control of motivational responses (Block and Block, 1980). Furthermore, impulsive and inhibitory behaviors may, in fact, be two independent constructs. Effortful control refers to one’s ability to voluntarily focus on and divert one’s attention from a situation, as well as to both inhibit and activate their control as appropriate (Valiente et al., 2003). Reactive control, as defined by Eisenberg et al. (2013), is a relatively involuntary or automatic type of control and can manifest as both reactive overcontrol (e.g., demonstrating inhibitions toward novelty) and reactive undercontrol (e.g., impulsivity). Reactive control, reactive undercontrol, and reactive overcontrol are considered three separate control-related constructs (Eisenberg et al., 2013). Moderating effects may often be present when researchers identify a relationship between two variables that is inconsistent with previous research (Bradley and Corwyn, 2002). Future research should explore moderating variables (e.g., parenting styles) between impulsive behavior and inhibitory behavior to deepen our understanding of their relationship.

The results of the present study also showed that the EUC-C had acceptable reliability and test–retest reliability. The two subscales—impulsive behavior and inhibitory behavior—also had acceptable reliability and test–retest reliability. These results support the validity of the EUC-C for studying ego-control among Chinese college students. The present results showed that the two subscales seem to be independent. Moreover, both theoretically and empirically, the global EUC-C scores among the Chinese college students showed the most stable validity compared to other EUC measures. The findings of the current study suggest that using the EUC-C scores for both impulsive behavior and inhibitory behavior to represent ego-control may better reflect that both undercontrol and overcontrol are undesirable, thereby highlighting the strengths of ego-control theory. Therefore, we recommend that future research utilize the total EUC-C scores to advance the study of ego-control. Of course, if a researcher is interested in exploring impulsive or inhibited behaviors, they may also collect data using the EUC-C and its subscale dimensions for related research, which may help us better comprehend the differences between these two behavior types.

Further studies should be conducted to better understand the role of ego-control within the Chinese cultural context. Consistent with our hypothesis, the results showed that ego-undercontrol was significantly positively associated with loneliness and depression and significantly negatively associated with self-esteem and life satisfaction. These results are in accordance with previous research (Letzring et al., 2005; Syed et al., 2020). This reflects the fact that ego-undercontrollers may experience various psychological maladjustments (e.g., low self-esteem). This may be because ego-undercontrollers have a low threshold for reactive patterns, display spontaneity, tend to directly express their needs and impulses, tend to immediately satisfy desires, and are prone to emotional and mood swings (Block and Block, 1980). Thus, it is not surprising that the ego-undercontrol is associated with maladjustment (Shiner, 1998).

### Limitations and future directions

There are some limitations to the current study that need to be addressed. First, the present study focused on the Chinese cultural context, which may limit the generalizability of the findings. Future research should explore whether these dimensions can be found to the same extent in other cultural contexts. Second, data from this study were collected from a sample of college students, raising questions about generalizability to other age groups. It is therefore important to validate the EUC-C in other samples (e.g., adolescents). Finally, further research is needed to explain the underlying mechanisms of the association between ego-control and psychological adjustment indicators, as well as the potential moderating factors of these associations.

There is currently a lack of measures assessing ego-control in the Chinese cultural context. The current study’s results support the EUC-C as a promising tool for measuring ego-control, undercontrol, and overcontrol in a Chinese context. As Block (2002) stated, the potential roles and applicability of the EUC are quite broad, as ego-control is conceptualized as the organizational structure of the personality system. Researchers studying self-control, self-regulation, self-depletion, delayed gratification, and other related areas may find that the EUC can be particularly useful (Letzring et al., 2005). For example, research has found that too much self-control (i.e., overcontrol) potentiates risk (Gilbert et al., 2020; Gilbert et al., 2022). Therefore, future research should investigate the mechanisms through which ego-control influences mental health, as well as the characteristics of the relationship between ego-control and self-control.

## Data Availability

Publicly available datasets were analyzed in this study. This data can be found here: The data that support the findings of this study are available upon request from the corresponding author. The data are not publicly available due to privacy and ethical restrictions.
