# Supplementary material for: Psychometric validation of the Ego-undercontrol scale in Chinese college students
Source: Front Psychol. 2025 Sep 11;16:1597994. doi: 10.3389/fpsyg.2025.1597994 (PMC12460088; doi:10.3389/fpsyg.2025.1597994)
Supplement: Supplementary file 1 [file Supplementary_file_1.docx]

**Supplementary Table 1**

Values of ITC and CR

| Item | ICT | CR |
| --- | --- | --- |
| 1. I tend to buy things on impulse. | .46^***^ | 19.40^***^ |
| 2. I become impatient when I have to wait for something. | .48^***^ | 20.75^***^ |
| 3. I often say and do things on the spur of the moment, without stopping to think. | .56^***^ | 22.64^***^ |
| 4. I can remember “playing sick” to get out of something. | .46^***^ | 19.35^***^ |
| 5. I have often had to take orders from someone who did not know as much as I did. | .39^***^ | 14.67^***^ |
| 6. When I get bored, I like to stir up some excitement. | .47^***^ | 18.05^***^ |
| 7. Some of my family have quick tempers. | .48^***^ | 19.25^***^ |
| 8. People consider me a spontaneous, devil-may-care person. | .51^***^ | 21.00^***^ |
| 9. I often get involved in things I later wish I could get out of. | .48^***^ | 18.25^***^ |
| 10. I have been known to do unusual things on a dare. | .43^**^ | 15.64^***^ |
| 11. I have sometimes stayed away from another person because I thought I might do or say something that I might regret afterwards. | .41^***^ | 14.13^***^ |
| 12. I do not always tell the truth. | .47^***^ | 17.52^***^ |
| 13. My way of doing things can be misunderstood or bother others. | .48^***^ | 18.62^***^ |
| 14. Sometimes I rather enjoy going against the rules and doing things I am not supposed to. | .59^***^ | 26.10^***^ |
| 15. At times, I am tempted to do or say something that others would think inappropriate. | .58^***^ | 24.39^***^ |
| 16. At times I have very much wanted to leave home. | .52^***^ | 21.78^***^ |
| 17. I would like to be a journalist. | .30^***^ | 12.24^***^ |
| 18. I like to flirt. | .39^***^ | 16.30^***^ |
| 19. Some of my family have habits that bother and annoy me very much. | .48^***^ | 19.42^***^ |
| 20. At times I have worn myself out by undertaking too much. | .44^***^ | 17.21^***^ |
| 21. In a group of people I would not be embarrassed to be called on to start a discussion or give an opinion about something I know well. | .19^***^ | 5.15^***^ |
| 22. I would like to wear expensive clothes. | .37^***^ | 15.82^***^ |
| 23. I am against giving money to beggars. (reverse scored) | –.15^***^ | –5.33^***^ |
| 24. It is unusual for me to express strong approval or disapproval of the actions of others. (reverse scored) | –.04^†^ | –0.86 |
| 25. I like to stop and think things over before I do them. (reverse scored) | .35^***^ | 13.10^***^ |
| 26. I don’t like to start a project until I know exactly how to proceed. (reverse scored) | .32^***^ | 11.07^***^ |
| 27. I finish one activity or project before starting another. (reverse scored) | .27^***^ | 9.88^***^ |
| 28. I am steady and planful rather than unpredictable and impulsive. (reverse scored) | .48^***^ | 19.72^***^ |
| 29. On the whole, I am a cautious person. (reverse scored) | .41^***^ | 15.98^***^ |
| 30. I do not let too many things get in the way of my work. (reverse scored) | .34^***^ | 13.05^***^ |
| 31. I keep out of trouble at all costs. (reverse scored) | .09^***^ | 2.77^**^ |
| 32. I consider a matter from every viewpoint before I make a decision. (reverse scored) | .39^***^ | 14.58^***^ |
| 33. I am easily downed in an argument. (reverse scored) | .01 | 0.42 |
| 34. I have never done anything dangerous for the fun of it. (reverse scored) | .39^***^ | 15.41^***^ |
| 35. My conduct is largely controlled by the customs of those about me. (reverse scored) | .05^*^ | 1.96^†^ |
| 36. It makes me uncomfortable to put on a stunt at a party even when others are doing the same sort of thing. (reverse scored) | .02 | 1.07 |
| 37. I find it hard to make small talk when I meet new people. (reverse scored) | .03 | 0.71 |

*Note:* ITC: item-total correlation.

CR: *t* values in independent sample *t* test of item discriminability analysis.

^†^*p*< .1, **p*<.05, ***p*<.01, ****p*<.001.

| **Supplementary Table 2**  Fit Indices of Exploratory Structural Equation Modeling Analysis of the Ego-undercontrol Scale | | | | | | | |
| --- | --- | --- | --- | --- | --- | --- | --- |
| Model | χ^2^ | *df* | *p* | CFI | TLI | SRMR | RMSEA [90% CI] |
| One-factor | 4841.669 | 377 | <.001 | .508 | .470 | .130 | .114 [.111, .117] |
| Two-factor | 1694.252 | 349 | <.001 | .852 | .828 | .046 | .065 [.062, .068] |
| Three-factor | 1347.729 | 322 | <.001 | .887 | .858 | .039 | .059 [.056, .062] |
| Four-factor | 989.313 | 296 | <.001 | .924 | .895 | .031 | .051 [.047, .054] |

| **Supplementary Table 3**  Standardized Factor Loadings for the One-, Two-, Three-, and Four-Factor Models | | | | | | | | | | | | | |
| --- | --- | --- | --- | --- | --- | --- | --- | --- | --- | --- | --- | --- | --- |
| Item | One-factor |  | Two-factor | |  | Three-factor | | |  | Four-factor | | | |
| 1 | **.48^*^** |  | **.47^*^** | .15**^*^** |  | **.53^*^** | .13**^*^** | –.16**^*^** |  | **.48^*^** | –.01 | .12**^*^** | .09 |
| 2 | **.59^*^** |  | **.59^*^** | .03 |  | **.66^*^** | .01 | –.18**^*^** |  | **.58^*^** | .01 | –.003 | .12**^*^** |
| 3 | **.59^*^** |  | **.57^*^** | .25**^*^** |  | **.62^*^** | .23**^*^** | –.14**^*^** |  | **.64^*^** | .08 | .20**^*^** | –.05 |
| 4 | **.47^*^** |  | **.47^*^** | .06 |  | **.47^*^** | .05 | .01 |  | **.30^*^** | .17**^*^** | .04 | .09 |
| 5 | **.44^*^** |  | **.44^*^** | .01 |  | **.51^*^** | –.01 | –.21**^*^** |  | **.44^*^** | –.09 | –.01 | .17**^*^** |
| 6 | **.56^*^** |  | **.56^*^** | –.04 |  | **.47^*^** | –.04 | **.31^*^** |  | .11**^*^** | **.52^*^** | –.07**^*^** | .07 |
| 7 | **.53^*^** |  | **.54^*^** | –.04 |  | **.55^*^** | –.05 | –.04 |  | .15**^*^** | –.002 | .003 | **.54^*^** |
| 8 | **.57^*^** |  | **.55^*^** | .21**^*^** |  | **.52^*^** | .21**^*^** | .10 |  | **.40^*^** | **.33^*^** | .16**^*^** | –.09**^*^** |
| 9 | **.60^*^** |  | **.60^*^** | –.01 |  | **.64^*^** | –.03 | –.09 |  | **.55^*^** | .13**^*^** | –.06 | .04 |
| 10 | **.47^*^** |  | **.48^*^** | –.07**^*^** |  | **.34^*^** | –.05 | **.43^*^** |  | .01 | **.66^*^** | –.11**^*^** | –.09 |
| 11 | **.57^*^** |  | **.59^*^** | –.12**^*^** |  | **.60^*^** | –.13**^*^** | –.01 |  | **.43^*^** | .20**^*^** | –.16**^*^** | .07 |
| 12 | **.56^*^** |  | **.57^*^** | –.03 |  | **.55^*^** | –.04 | .06 |  | **.31^*^** | .24**^*^** | –.05 | .12**^*^** |
| 13 | **.59^*^** |  | **.58^*^** | .07**^*^** |  | **.58^*^** | .06 | .02 |  | **.43^*^** | .24**^*^** | .02 | .01 |
| 14 | **.66^*^** |  | **.65^*^** | .14**^*^** |  | **.51^*^** | .16**^*^** | **.44^*^** |  | .05 | **.69^*^** | .12**^*^** | .07 |
| 15 | **.70^*^** |  | **.70^*^** | .04 |  | **.63^*^** | .05 | .20**^*^** |  | .24**^*^** | **.43^*^** | .03 | .17**^*^** |
| 16 | **.61^*^** |  | **.61^*^** | –.02 |  | **.57^*^** | –.02 | .11 |  | .03 | .18**^*^** | .03 | **.58^*^** |
| 17 | .27^*^ |  | .27**^*^** | .02 |  | .22**^*^** | .02 | .15**^*^** |  | –.03 | .21**^*^** | .03 | .15**^*^** |
| 18 | **.38^*^** |  | **.38^*^** | .01 |  | .29**^*^** | .02 | .26**^*^** |  | –.04 | **.36^*^** | .01 | .16**^*^** |
| 19 | **.56^*^** |  | **.57^*^** | –.06 |  | **.56^*^** | –.06 | .03 |  | –.02 | –.002 | .02 | **.79^*^** |
| 20 | **.52^*^** |  | **.54^*^** | –.131**^*^** |  | **.52^*^** | –.14**^*^** | .05 |  | .08 | .12**^*^** | –.10**^*^** | **.48^*^** |
| 22 | **.40^*^** |  | **.41^*^** | –.08**^*^** |  | **.37^*^** | –.07**^*^** | .13**^*^** |  | .02 | .20**^*^** | –.06 | .29**^*^** |
| 25 | .05 |  | –.03 | **.76^*^** |  | –.06 | **.76^*^** | –.001 |  | .02 | .01 | **.75^*^** | –.10**^*^** |
| 26 | –.012 |  | –.09**^*^** | **.67^*^** |  | –.15**^*^** | **.68^*^** | .10**^*^** |  | –.19**^*^** | .06 | **.68^*^** | –.01 |
| 27 | –.05 |  | –.12**^*^** | **.59^*^** |  | –.19**^*^** | **.60^*^** | .14**^*^** |  | –.26**^*^** | .09**^*^** | **.61^*^** | .01 |
| 28 | .20**^*^** |  | .13**^*^** | **.80^*^** |  | .11**^*^** | **.80^*^** | –.01 |  | .06 | .01 | **.80^*^** | .05 |
| 29 | .13**^*^** |  | .05 | **.77^*^** |  | .04 | **.77^*^** | –.03 |  | .07 | –.01 | **.76^*^** | –.04 |
| 30 | .06 |  | –.01 | **.69^*^** |  | –.004 | **.68^*^** | –.09**^*^** |  | –.02 | –.14**^*^** | **.71^*^** | .12**^*^** |
| 32 | .08**^*^** |  | .004 | **.77^*^** |  | .01 | **.77^*^** | –.10**^*^** |  | .08**^*^** | –.11**^*^** | **.77^*^** | –.01 |
| 34 | .16**^*^** |  | .13**^*^** | **.35^*^** |  | .02 | **.36^*^** | **.30^*^** |  | –.21**^*^** | **.34^*^** | **.35^*^** | .02 |

*Note:* Items with loadings |≥.30| are bolded.

**p*<.05.

**Supplementary Table 4**

Fit Indices of Exploratory Structural Equation Modeling Analysis of the Chinese Version of the Ego-undercontrol *Scale (*EUC-C*)*

| Model | χ^2^ | *df* | *p* | CFI | TLI | SRMR | RMSEA [90% CI] |
| --- | --- | --- | --- | --- | --- | --- | --- |
| One-factor | 1086.196 | 44 | <.001 | .730 | .662 | .130 | .161 [.153, .170] |
| Two-factor | 157.024 | 34 | <.001 | .968 | .948 | .025 | .063 [.053, .073] |

**Supplementary Table 5**

Fit Indexes for Measurement Equivalence Tests of Ego-undercontrol Across Gender

| Model | χ^2^ | *df* | CFI | TLI | SRMR | RMSEA[90% CI] | Model comparison | △CFI | △TLI | △RMSEA |
| --- | --- | --- | --- | --- | --- | --- | --- | --- | --- | --- |
| Model 1 | 457.644^***^ | 86 | .952 | .938 | .042 | .069 [.063, .075] |  |  |  |  |
| Model 2 | 473.685^***^ | 95 | .951 | .943 | .046 | .066 [.06, .072] | 2 vs 1 | –.001 | .006 | –.003 |
| Model 3 | 513.716^***^ | 104 | .947 | .944 | .046 | .066 [.06, .072] | 3 vs 2 | –.005 | .001 | 0 |

*Note*: Model 1 = Configural Invariance; Model 2 = Metric Invariance; Model 3 = Scalar Invariance.

^***^*p*< .001.

**Supplementary Figure 1**

Scree Plot With The Parallel Analysis Line


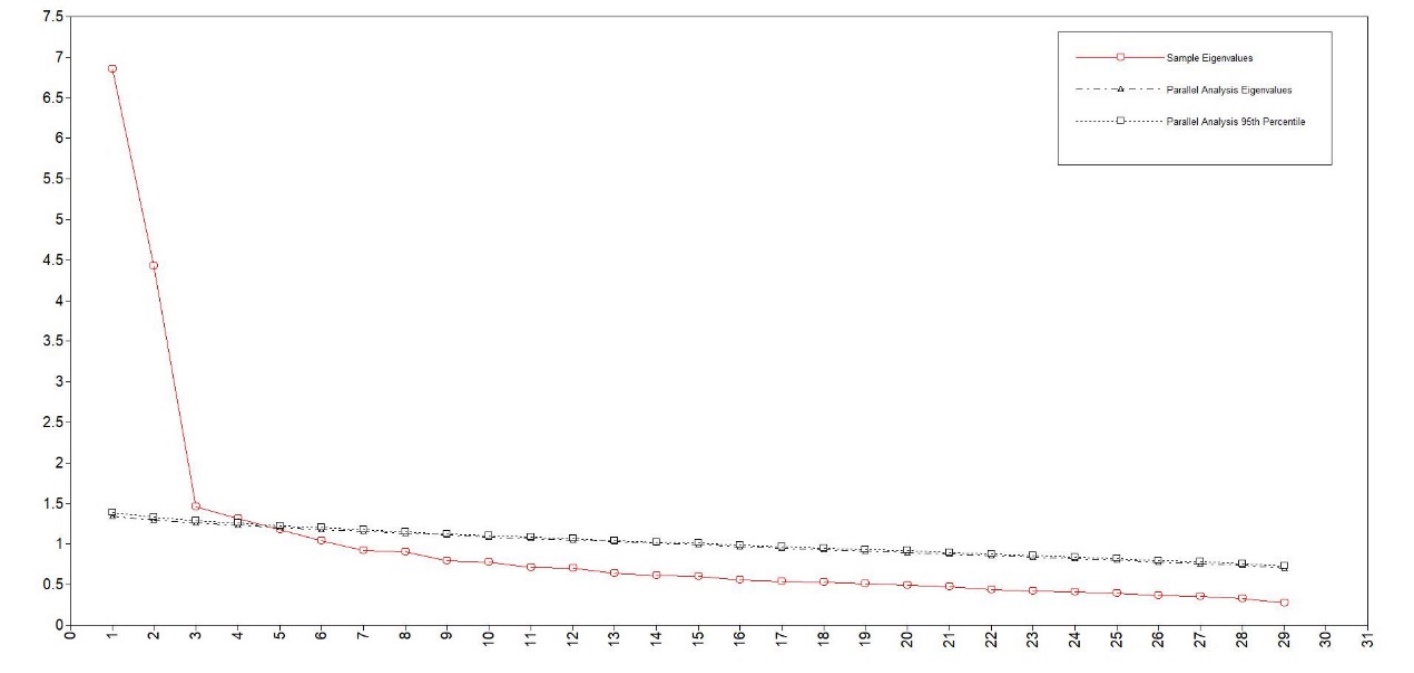


Eigenvalue

Factor

**Supplementary Figure 2**

Standardized Factor Loadings for the Chinese Version of the Ego-undercontrol Scale


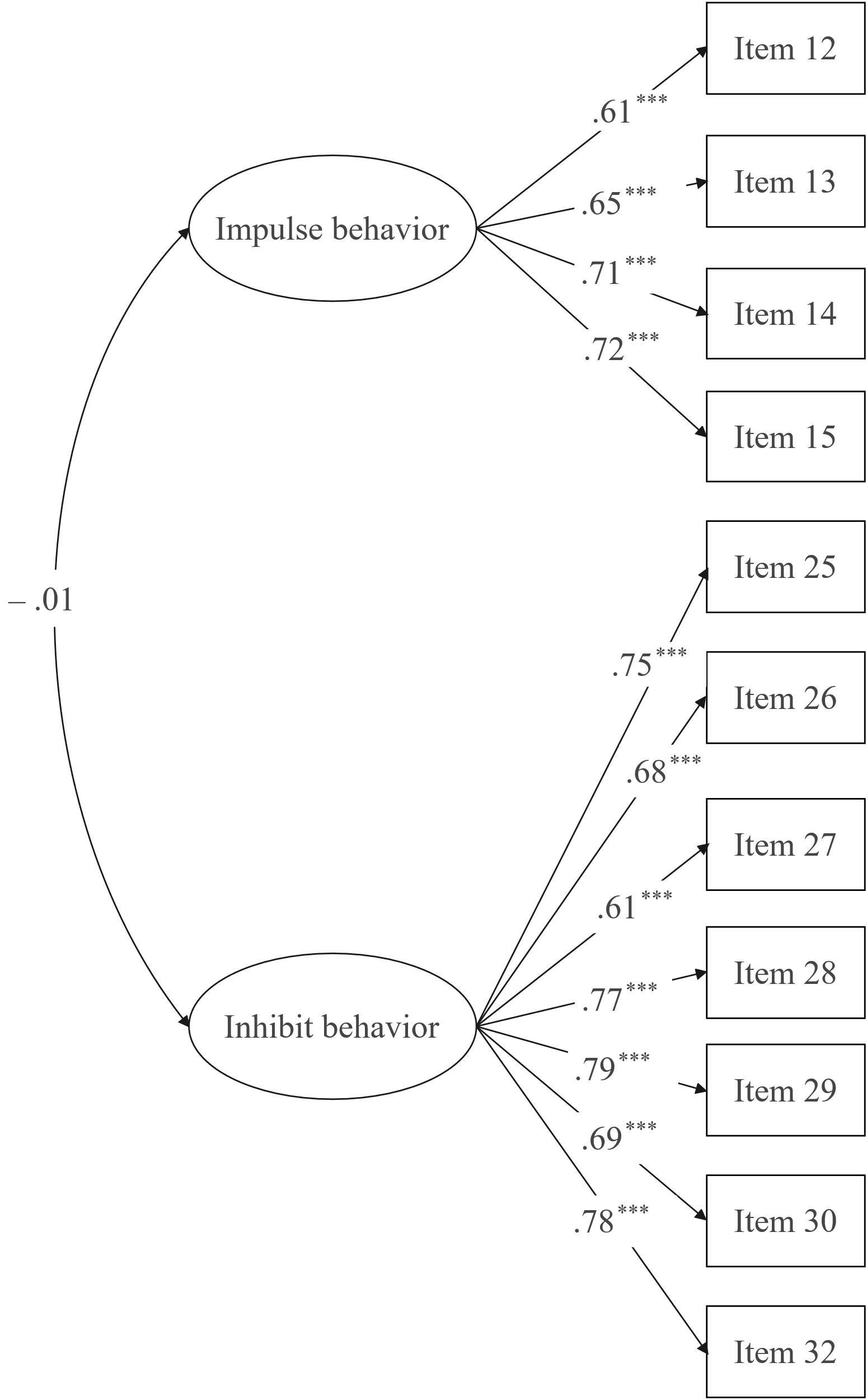


*Note: ***p<.001.*
